# A Novel Sensor Platform Matching the Improved Version of IPMVP Option C for Measuring Energy Savings

**DOI:** 10.3390/s130506811

**Published:** 2013-05-22

**Authors:** Yen-Chieh Tseng, Da-Sheng Lee, Cheng-Fang Lin, Ching-Yuan Chang

**Affiliations:** 1 Graduate Institute of Environmental Engineering, National Taiwan University, No. 1, Sec. 4, Roosevelt Road, Taipei 10617, Taiwan; E-Mails: yenj36@gmail.com (Y.-C.T.); cflin@ntu.edu.tw (C.-F.L.); 2 Department of Energy and Refrigerating Air-Conditioning Engineering, National Taipei University of Technology, No. 1, Sec. 3, Chung-Hsiao E. Rd., Taipei City 106, Taiwan; E-Mail: f11167@ntut.edu.tw

**Keywords:** sensor platform, IPMVP, energy savings, EPC global architecture framework, vertical garden system (VGS), environmental monitoring

## Abstract

It is easy to measure energy consumption with a power meter. However, energy savings cannot be directly computed by the powers measured using existing power meter technologies, since the power consumption only reflects parts of the real energy flows. The International Performance Measurement and Verification Protocol (IPMVP) was proposed by the Efficiency Valuation Organization (EVO) to quantify energy savings using four different methodologies of A, B, C and D. Although energy savings can be estimated following the IPMVP, there are limitations on its practical implementation. Moreover, the data processing methods of the four IPMVP alternatives use multiple sensors (thermometer, hygrometer, Occupant information) and power meter readings to simulate all facilities, in order to determine an energy usage benchmark and the energy savings. This study proposes a simple sensor platform to measure energy savings. Using usually the Electronic Product Code (EPC) global standard, an architecture framework for an information system is constructed that integrates sensors data, power meter readings and occupancy conditions. The proposed sensor platform is used to monitor a building with a newly built vertical garden system (VGS). A VGS shields solar radiation and saves on energy that would be expended on air-conditioning. With this platform, the amount of energy saved in the whole facility is measured and reported in real-time. The data are compared with those obtained from detailed measurement and verification (M&V) processes. The discrepancy is less than 1.565%. Using measurements from the proposed sensor platform, the energy savings for the entire facility are quantified, with a resolution of ±1.2%. The VGS gives an 8.483% daily electricity saving for the building. Thus, the results show that the simple sensor platform proposed by this study is more widely applicable than the four complicated IPMVP alternatives and the VGS is an effective tool in reducing the carbon footprint of a building.

## 1. Introduction

During the past two decades, primary energy consumption has grown by 49% and CO_2_ emissions by 43%, with an average annual increase of 2% and 1.8%, respectively. Current predictions show that this growth will continue. The energy used by nations with emerging economies will grow at an average annual rate of 3.2% and will exceed that for developed countries by 2020, with an average growth rate of 1.1%. In particular, mainland Asia's energy consumption will continue to grow at an average rate of 3.7%. As a response to the challenge of climate change, several research and modeling exercises have been performed, mostly discussing options such as a shift towards low carbon fuels, renewable energy and improvements in the efficiency of energy transformation and end-use technologies. However, engineering cannot provide all of the solutions; cultural and behavioral change that alters the demand for energy services is necessary in order to meet the stringent targets for climate change mitigation [1]. A reduction in energy-service demands will, *ceteris paribus*, result in lower energy consumption and a consequent reduction in CO_2_ emissions for the entire energy system [2]. The development of energy saving technologies to suppress reduce energy use is an effective way to overcome the threat of climate change.

The improvement of a building's envelope and the energy consumption patterns of humans represent the two most feasible methods of increasing energy efficiency and reducing gas emissions. China reduced its energy consumption by one third, during the period 1978–1990, simply by employing elementary design and construction technologies [3].

Electricity consumption for the needs of everyday life accounts for 20 percent of total electricity consumption in Taiwan. Other users of electricity, such as the industrial and commercial sectors, petrochemical, steel, cement, paper and man-made fiber industries, account for 25% of Taiwan's total electricity consumption. In the year 2000, the electricity demand in Taiwan was 2,042.6 billion kWh, and this figure is estimated to increase to 2,759.8 billion kWh by 2019. The annual average growth rate is 3.4%, so an extra 7.2 billion kWh of electricity is required, annually, in order to meet the electricity requirements for future economic development [4]. Information technology-enabled monitoring and control systems can assist in mitigating energy use in residences, by more efficiently allocating the delivery of services, in terms of time and location. A great deal of energy is wasted in the inefficient delivery of services to residents, for example the heating or cooling of unoccupied spaces, overheating/undercooling for whole-house comfort, leakage current and inefficient appliances. The initial estimate is that more than 39% of residential primary energy is wasted [5]. Energy saving is an important issue, as energy prices increase. Products and services provided by the information and communication technology (ICT) sectors allow greater energy efficiency and the emission of reductions. The use of ICT in a residential area results in a smart building environment. Ecological and smart building networks should not only improve the quality of human life in terms of health care, security and the efficient management of energy and resource consumption, but should also provide convenience and personal fulfillment, through easy communication with others in the community, sophisticated entertainment options and opportunities for continuous education. A smart building in a smart grid represents the latest evolution of the principle of ICT usage in a building [6].

A wireless sensor network is composed of a large number of tiny sensor nodes [7]. The main task of a wireless sensor node is to sense and collect data from a certain area, process these data and transmit them to a sink node, where further processing of the collected data can be performed [8]. Sensor nodes are small-scale and cost effective devices with limited capabilities. Wireless sensor networks generally contain thousands of sensor nodes, randomly deployed in a field. The sensor nodes are powered by batteries and controlled remotely. For many applications, the recharging or replacement of these batteries in the sensor nodes after deployment is impossible [9].

Depending on the use of sensor networks, certain routing protocols are required, in order to allow communication between sensor nodes and the sink nodes [10]. Wireless sensor networks consume a limited amount of energy in collecting data, performing calculations and routing the received data, so in most applications, each sensor node is expected to last for a long time [11]. For this reason, both the efficient use of energy and efficient routing schemes are highly important in sensor networks [9].

**Figure 1.**
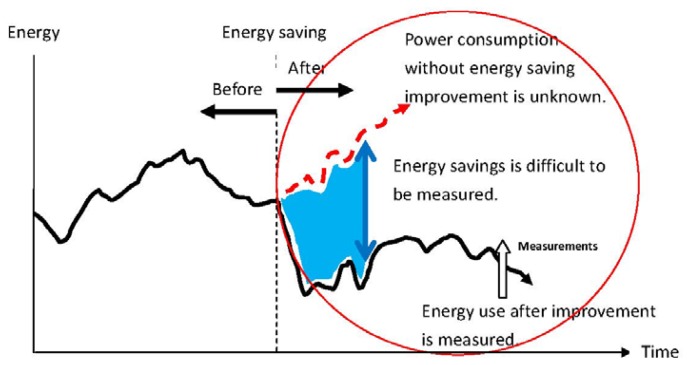
Before and after energy saving improvements, energy use is easy to measure and track, but energy savings cannot be measured, because the power consumption without those improvements cannot be determined.

Published research has demonstrated effective ways of cutting energy use, through the development of new technology. If this strategy is successful, a reduction in the growth in energy consumption, as discussed in previously, can be achieved. However, before these new techniques can be practically applied, one technical issue must first be addressed, in that there must exist a means to quantitatively measure energy savings. Although power consumption before and after energy saving improvements are implemented can be tracked, the actual amount of energy saved is hard to determine. Figure 1 illustrates the problem.

As illustrated in Figure 1, energy savings are difficult to measure, because the power consumption without the energy saving improvement is impossible to measure. Thus, most research related to the development of energy saving technology uses a comparison method. Energy use before and after applying the energy saving techniques is measured and compared, to determine the energy saved. The assumption is that the energy use before and after the improvements is the same. In the laboratory, conditions can be adjusted to concur with this assumption and accurate energy savings can be calculated. The comparison method works experimentally, but in real applications, where such adjustments are not possible, the energy savings reported by assuming unchanged energy use are not correct. A tool for measuring energy savings must report those savings by measurement. This is a key requirement for research related to energy saving technologies. Only if the energy saving effects can be verified accurately, can energy saving research provide real commercial products that ensure a low carbon future.

In order to accurately estimate these energy savings, the internationally recommended International Performance Measurement and Verification Protocol (IPMVP) defines standard terms and suggests best practices for quantifying the results of energy efficiency investments and for increasing investment in energy and water efficiency, demand management and renewable energy projects [12]. The IPMVP was developed in 1994–1995 by a coalition of international organizations, led by the United States Department of Energy [13].

The Protocol has become the national measurement and verification standard in the United States and many other countries [13]. A major driving force for the standard was the need for a common protocol to verify savings claimed by Energy Service Companies (ESCOs), when implementing Energy Conservation Measures (ECMs). The protocol is a framework to determine the water and energy savings associated with ECMs. IPMVP provides four options for the determination of savings (identified as A, B, C and D) [14]. Options A and B focus on the performance of specific ECMs and involve measuring the energy use of systems affected by each ECM, separately from that of the rest of the facility. Before and after measurements are compared, to determine savings. A lighting retrofit and installation of variable speed drives are examples of options A and B. Options C and D assess the energy savings at the facility level, when the ECM cannot not easily measured in isolation from the rest of the building. Option C assesses savings by analyzing utility bills before and after the implementation of the ECM. Option D uses simulations of equipment or facilities, when base year or post-retrofit data are unreliable or unavailable [13]. These four options of A, B, C and D are recommended by the IPMVP to measure saved energy. However, every methodology has its limits in implementations, as indicated in Table 1.

Options C and D require more time and skill and are more costly to measure than options A and B [13]. Option C recommends a methodology to report saved-energy for the entire facility. By analyzing meter data, option C gives reliable evidence to show energy savings. It is a widely employed method, since it provides a maximum measurement boundary. However, this methodology has limited application, as the expected savings must exceed 10%. This restricts the range of application range for option C for most existing buildings to only an elementary saving project *i.e.* the energy saving effect cannot be less than 10% [12].

Option D is an alternative methodology with the same measurement boundary. It uses computer simulation software to predict the energy for the facility. A simulation model must be calibrated so that it predicts an energy pattern that approximately matches the actual metered data. However, simulated data must be interpreted by experts and some facilities may not have access to such expertise, so option D is of limited benefit to end users [12]. Therefore, all four IPMVP options have their limitations, especially those that cannot provide an actual system boundary for the section wherein savings are to be measure.

Conditions which vary in a predictable fashion are normally included within the basic mathematical model used for routine adjustments. Unexpected or one-time changes may require non-routine adjustments, normally called simple Baseline Adjustments. Examples of situations that often need Baseline Adjustments are: (i) changes in the amount of space being heated or air conditioned; (ii) changes in the amount or use of equipment; (iii) changes in environmental conditions (lighting levels, set-point temperatures, *etc.*) for the sake of standards compliance; and (iv), changes in occupancy, schedule, or throughput [12].

The facility can be a building or even an entire complex of buildings. Equipment is the machinery or tools inside the facility. In this study, option A and B only focus on equipment; option C and D consider the entire facility or building, but option C lacks accuracy.

## 2. Experimental Section

**Figure 2.**
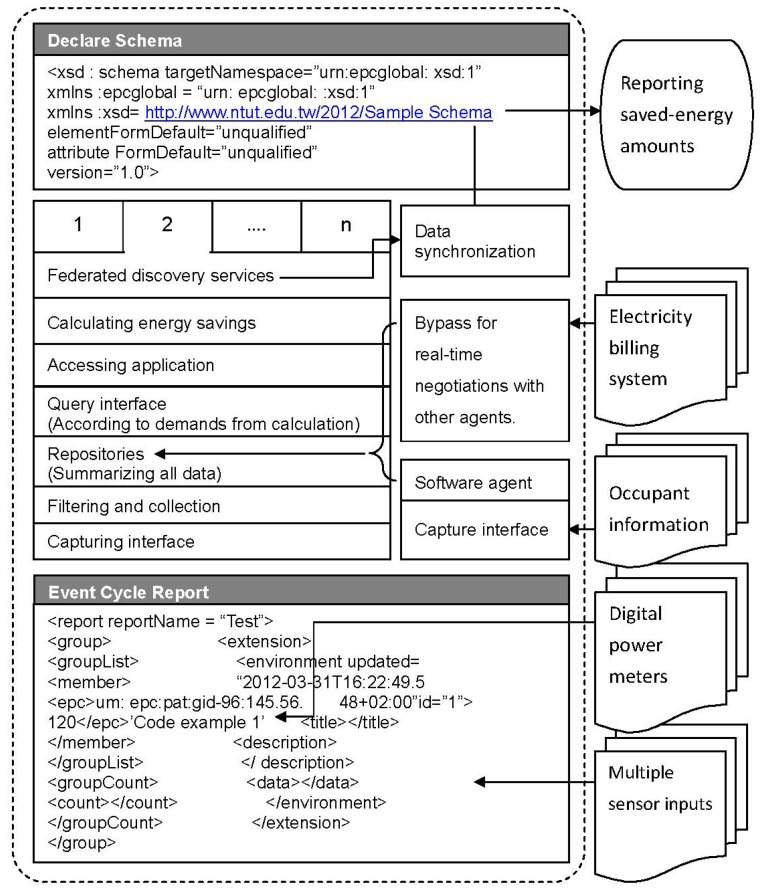
Architectural framework of the sensor platform for the measurement of energy savings.

In this study, a sensor platform is proposed for the measurement of energy savings. This platform composes distributed sensors; digital power meters and information systems, such as occupancy information and electricity billing systems. In order to process multiple data, an architectural framework is used, as shown in Figure 2. Figure 2 shows the information structure of the sensor network. The architectural framework shown in Figure 2 is based in the Electronic Product Code (EPC) global architecture framework [16]. Data is collected from multiple sensors and digital power meters, using event cycle reports. The architectural framework collects commercial wireless communication module tag information. In a supply chain, there are many tags, containing complicated commercial information. An event cycle provides a means to assign microprocessor data to applications, by specifying multiple data resources. This is the sensor platform for the measurement of energy savings. Multiple data from sensors and power meters must be analyzed and correlated. We use data to improve IPMVP option C to calculate the amount of energy saved.

An Extendable Markup Language (XML)-based program is used to gather data, using a group concept. The group core is a digital power meter record, identified by Global Identification (gid). As shown in Figure 2, the power meter readings link to <epc><um: epc:pat:gid-96:145.56.48+02:00”id=”1”></epc>. One gid number indicates the energy use in one specific location within the facility. One gid includes related members in an event cycle report. Using <extension>, multiple sensor inputs are attached at <data></data> line.

The capture interface is the sensor router hardware. The above program is executed on one sensor router to collect data from multiple sensors and then stratify the data, as described in the event cycle report. At the capture interface, filtering and collection, data is filtered and collected, according to the demands of the query interface. Information on occupants is also collected by the capture interface. Differently from that used for sensors, a software agent is needed to translate the data for different information systems and the private database. Data from the information system is sent to repositories, a buffer to store data. In conjunction with the billing data from a public database, such as the power plant billing system, all data is ready for calculation of the amount of energy saved.

By accessing the application, the query interface sends demands to the repositories, according to the requirements for the simulation of the entire facility and calculation of the energy consumption, with and without energy conservation measures. The functional block diagram for the calculation of energy savings has a related algorithm. According to this algorithm, data demands are determined and queried through the query interface discussed previously.

**Figure 3.**
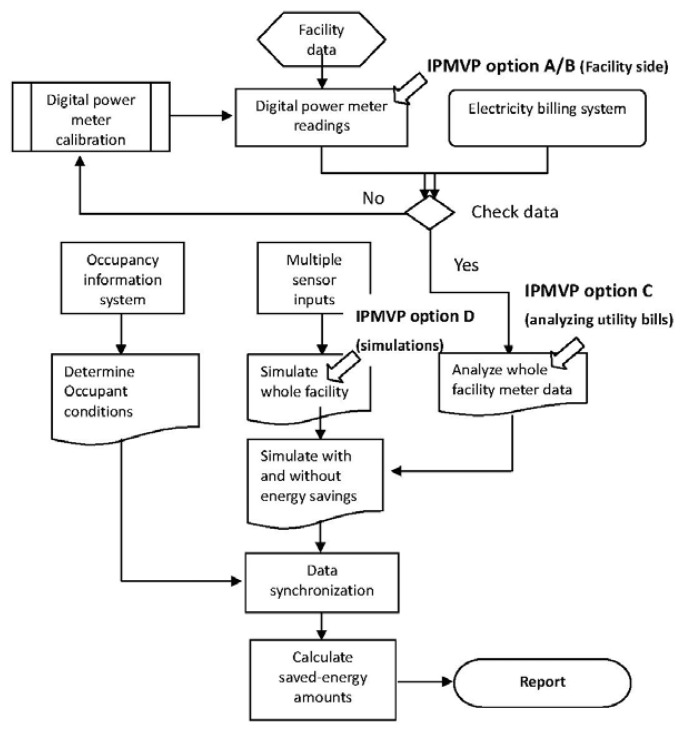
Algorithm for the sensor platform to determine the amount of energy saved.

All procedures are defined as a federated discovery service, according to the EPC global architectural framework. When all of the data has been synchronized, the amount of energy saved can be determined and reported using a declare schema. As illustrated at the top of Figure 2, the standards declare schema exports energy savings through the Internet. Appropriately qualified personnel can access the data and identify the energy savings. Figure 2 shows the information structure of the sensor network, the key block diagram shows the calculation of energy savings. The algorithm is detailed in Figure 3.

The sensor platform determines the energy savings, this study's Idea using combination of the four options of IPMVP. As the first step, facility data is input as the basis for determining the energy savings for the entire facility. Then, using options A/B, digital power meters are used to determine the savings (e.g., measure electricity consumption and the energy saving coefficient of an air conditioner), by field measurement of the energy used by the system with energy conservation measures. The detected amount of energy used is checked against the electricity bill. If these do not correspond, the meters are calibrated to ensure that they give accurate data.

As shown in Figure 3, option A/B only shows the savings on the equipment side. The sensor platform with an architectural framework can integrate all of the data to determine the energy use for the entire facility. As indicated in Figure 3, the digital power meter readings and information from electricity billing system are checked and combined as a single result. Using option C, a baseline for the entire facility is constructed.

In contrast to option C, the sensor platform not only analyzes power use data, but also uses the distributed sensors to simulate the entire facility. Multiple sensor inputs simulate the effect of a multifaceted energy management program on many systems in a facility. The simulation procedures are those of option D, but sensor data is used instead of a mathematical model.

The occupancy information for the entire facility is obtained from other information systems or databases, such as an “Attendance System” or a “Sign-in System”. The power meter readings, multiple sensor inputs and occupancy conditions are synchronized to determine the energy savings, in accordance with the following equation:

(1) $$\begin{array}{ll} \text{Savings} & =\text{Baseline energy form a hypothetical model}\left[\text{without energy savings}\right]\\ & -\text{Reporting}-\text{period energy from measured data}\left[\text{with savings}\right] \end{array}$$

The energy savings determined by Equation (1) depend entirely on the measured data. Even for baseline energy, the hypothetical model uses multiple sensor inputs, but infers the energy consumption without energy conservation measures from the sensing data. This algorithm is the key function of the block diagram—calculating energy savings, as shown in Figure 2. In order to illustrate the operation of the algorithm, a real case is presented. The platform was used to monitor a building with a newly built vertical garden system and the energy savings were determined using the sensor platform. The following section describes the energy conservation project. As well as the power meter and sensor data, the occupancy information is also collected by the sensor platform.

### 2.1. A Case Study for the Sensor Platform: A Vertical Garden System (VGS)

A Vertical Garden System (VGS) is different from a general plant wall in that it not only controls the plant growth area, but the vegetation can also be adjusted to a thickness and range that is appropriate to the demands of the building. The Center for Development of Low-Carbon, Green Energy and Eco-Community constructed a VGS in order to save energy, reduce carbon consumption and for environmental protection. The VGS is located at the junction of Chung Hsiao East Road and Xinsheng South Road, as a symbol of the Taipei University of Technology's desire to provide a beacon of ecological planning for urban buildings.

**Figure 4.**
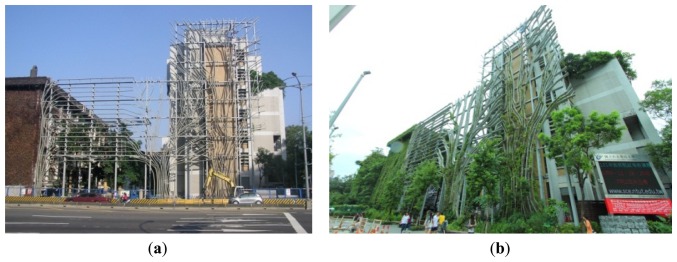
Case study: A building shielded by a vertical garden system (VGS) saves air-conditioning energy. (**a**) Structure of the VGS. (**b**) Climbing plants provide shade.

The VGS has an overall weight of 126,510 kg. The foundation material is cement, which provides stable support. The VGS is mainly constructed from fibre-reinforced plastic (FRP) materials, supported by steel (H-shaped steel). The appearance of building shielded by a VGS for the case study is shown in Figure 4.

#### 2.1.1. Experimental Apparatus (Sensor Configuration)

The wireless sensor nodes in the energy system collected physical information about the indoor environment, which is designed for human comfort. Three types of sensor network were used: dual-use temperature and humidity, illumination and wind speed sensors. The nodes were placed indoors and outdoors. The sensors, the signal processing, model, the measurement range and the power consumption of the wireless transmission modules and solar collector panels are shown in Table 2.

The signal from the sensor was processed by a microprocessor (PIC16F526) and data was transferred by TI CC1101 (from TI Co. Ltd., Dallas, TX, USA), combined into a commercial wireless communication module (PIC16F526 + TI CC1101).

The Cloud monitor platform used an Intelligent Energy Network-Application Service Provideri (IEN-ASP) system. iEN-ASP is a well-known network platform for energy saving control at Taiwan. This is provided by installing a computer monitoring device in the air-conditioning system, such as chiller, pre-cooling A/C box, air-conditioning unit, fan coil unit, water tower, pump and box-type A/C unit (package), window-type and split-type A/C, *etc.* Further, various environmental sensors will also be installed in the air-conditioning environment such as temperature sensor, humidity sensor, CO_2_ sensor and mobile sensor, *etc.*, to collect the A/C equipment operation and environment status data. The environment was continuously monitored by all of the sensors, and information collected by the sensors was sent back to the server via the Internet. All of the necessary information from a specific place was provided by the iEN-ASP platform [17].

#### 2.1.2. Sensor Node

**Figure 5.**
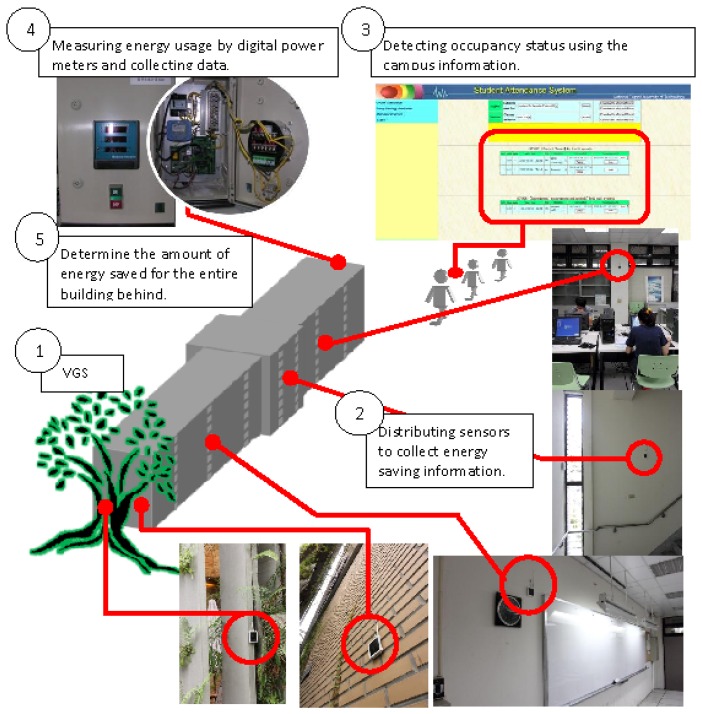
Sensor configuration.

Figure 5 illustrates the sensor configuration. Temperature and humidity, wind speed and illumination sensor network nodes used external power cords or batteries as power sources. However, power cables must be laid and batteries replaced or recharged, so a renewable energy source was sought.

Measurements were made at the building's interior and the exterior, including heat, vibration and ligh. The solar collector generated by the highest unit density of energy, from solar radiation stray light from indoor lighting [18].

In this study, the hygrometer and thermometer sensor used were the SHT10 in IC type. The component uses sintered ceramics packaged in a Complementary Metal–Oxide–Semiconductor (CMOS) chip. The ceramics adsorb moisture and change active capacitance to monitor the relative humidity. The CMOS support digital-analog is transformed from the back end. The digital data is sent using a Inter-Integrated Circuit (I2C) transmitter and the humidity is measured with ±4.5% precision, for 20% ∼ 80% relative humidity.

**Figure 6.**
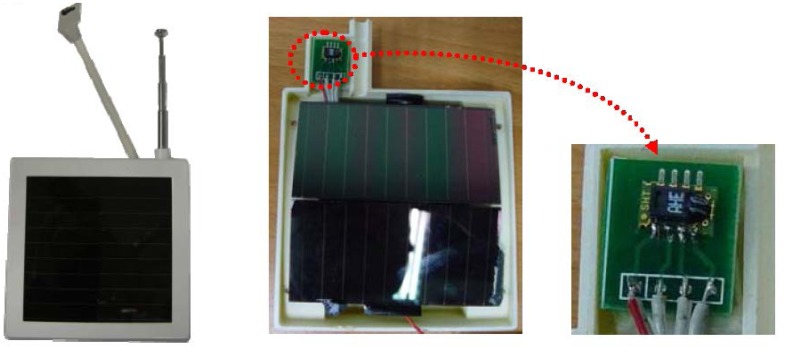
Sensor node.

The sensor provides a precise diurnal variation curve for measurements at −40 °C ∼ 100 °C and the range of relative humidity. Sensor's power consumed by the component was 30 summit and was connected with a microprocessor, without the need for peripheral filter circuits, because of the sensor's digital output. The wireless Temperature/Humidity sensor is shown in Figure 6 [18].

**Figure 7.**
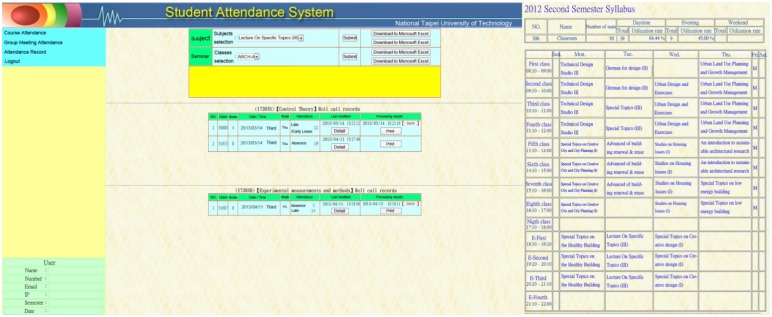
Special sensor interface—campus information system.

The number of people in the space was determined using the Auto Roll-Call System (see Figure 7), which records the number of people in each class and the use of space to determine the energy consumption for indoor/air conditioning heat load, to assess the effect of thermal insulation. In this study, the electricity-consumption and temperature were sent via a sensor and the smart saving energy system monitored the amount of energy used. All of the information was processed using options C and D of the IPMVP, to simulate and test whether a real could produce comparable energy savings.

### 2.2. Uncertainty Analysis of IPMVP Option C

The amount of energy savings detectable using option C is limited to 10%, so if the energy saved is less than 10%, it is not noted. This uncertainty is critical and the system can be upgraded at an extra 25% cost, which is an expensive solution [12]. This uncertainty in energy savings is attributed to modeling errors and an instrumentation errors. A modeling error refers to errors in the models used to estimate the parameters of interest from the data collected. Biases in these models arise from model mis-specification. This includes the assignation of incorrect values for known factors and the extrapolation of the model's results outside their range of validity. The magnitude of the instrumentation errors is given by the manufacturer's specifications. Typically, instrumentation errors are small and are not the major source of error in estimating savings. The uncertainty is caused by investigation outside the boundary; for example, an external environment, or early termination of a test, or non-constant variation of a parameter. The energy consumption data may change, since other device may interfere with energy saving measures. Non-systematic errors occur because of the random effects of factors not accounted for by the model variables. The most common models are linear regressions of the Equation (2):

(2) $$y=b_{0}+b_{1}x_{1}+b_{2}x_{2}+\ldots b_{\text{p}}x_{\text{p}}+c$$

In this formula, y and x_k_, k = 1, 2, 3 … p monitors the variable; b_k_, k = 0, 1, 2 … p regresses the estimated coefficient and e is the residual sum which cannot be regressed to the equation.

This study addresses the 10% limitation of option C, by reducing the uncertainty and providing energy saving in a real case.

### 2.3. IPMVP Options A, B, C and D Versus an Improved Version of Option C

The comparisons of IPMVP options and improved version of option C are shown in Table 3 [12]. The results illustrate that an improved version of option C is simple to use and more applicable.

## 3. Results and Discussions

**Figure 8.**
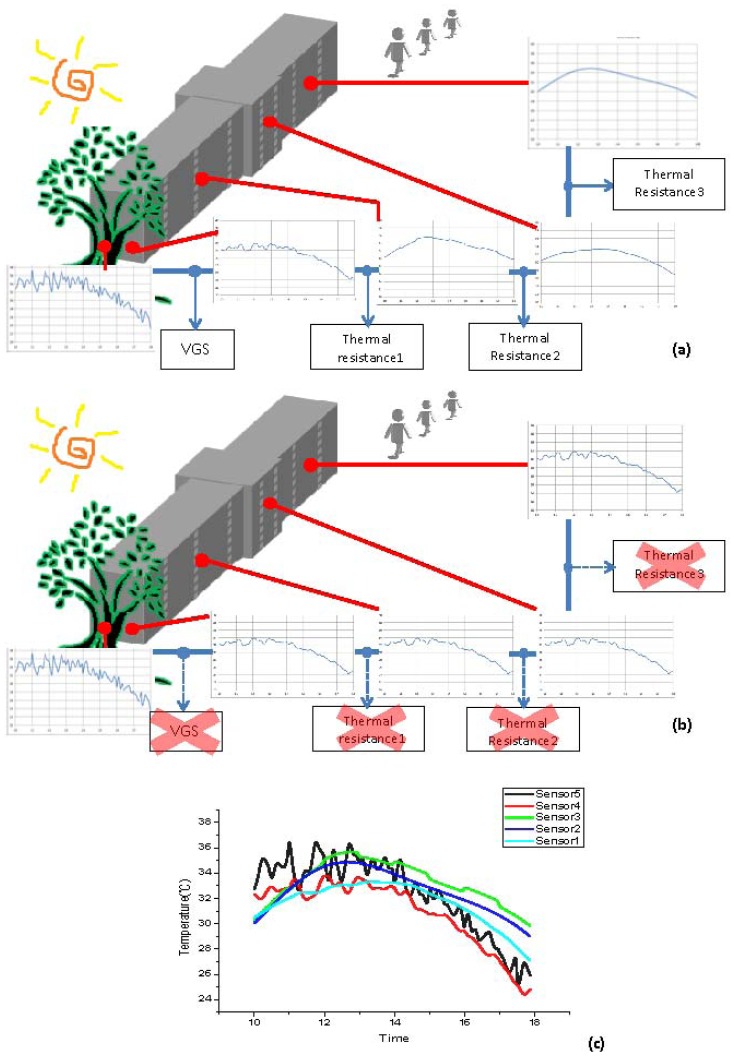
System model.

The increasing number of buildings means that greener architecture is required, however the improvements provided by this strategy must be quantified. The sensor network on the VGS monitored the effect of green vegetation and also allowed determination of the energy savings for the building. Cloud management was used to monitor the VGS using sensors in the building. The VGS was placed on the west side of the building, to reduce the amount of air from outdoors. The thermal resistance system is shown in Figure 8(a). The building faces west in the afternoon and since VGS blocks the outdoor air the resistance increases and there is a smoothing of the changes to the indoor temperature. Without VGS, the indoor temperature is not stable, as seen in Figure 8(b).

**Figure 9.**
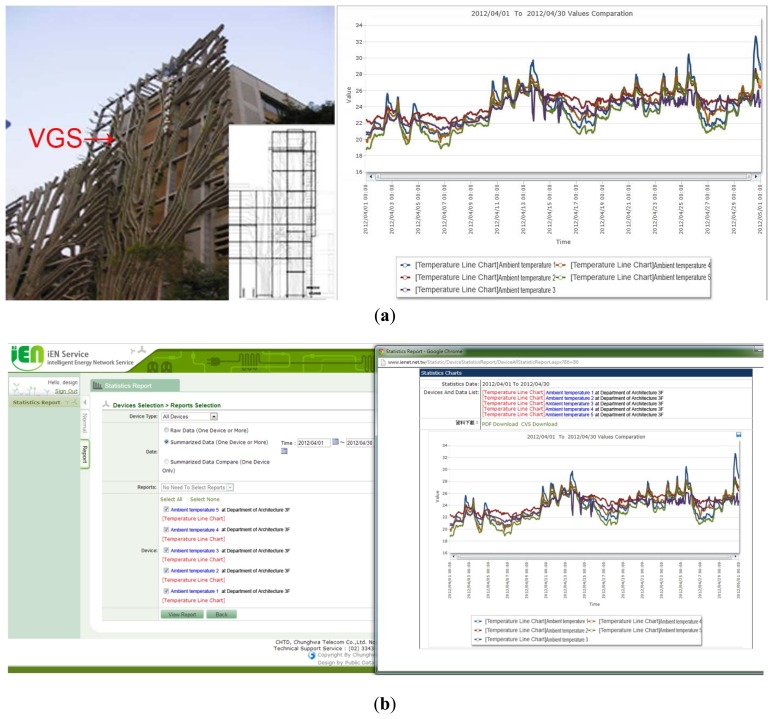
VGS and cloud management system (user interface of iEN-ASP).

Figure 8(c) shows that the temperature change at the green gate (sensor 1) is larger than the indoor temperature change. The VGS provides more thermal resistance and makes the temperature lower and the temperature change smaller. The green gate stabilizes the temperature change and also reduces the temperature. The data and from environmental monitoring were captured using a remote platform. The cloud system monitored the building's energy usage. The cloud management control interface is shown in Figure 9.

### 3.1. A Comparison with Actual Measured Data

In this study we have screened out days with the same atmospheric environment for analysis. Our temperature data collection lasted for nine months (from October 2011 to June 2012). Out of a sample size of 274 days, the authors have picked 10 days with a similar atmospheric environment. The weekday temperatures of classrooms located at the east side of the building were taken to be the basic temperatures of a classroom (without VGS) and the indoor temperatures were monitored with air conditioners working. Those located at the west of the building were taken to be the energy saving temperatures of a classroom (with VGS) and the indoor temperatures were monitored with air conditioner working. On weekends and winter and summer breaks, the temperatures of classrooms were taken without air conditioners working on both the east and west sides.

The east side of the building was in its original form without a VGS while the west side had the VGS. Therefore, after a day's exposure to the Sun, this allowed the authors to compare the temperatures of the east and west sides and verify the energy saved. For the ten days the authors have screened out, the west side's (with VGS) average temperature was 22.275 °C, and the east side without VGS had an average recorded temperature of 23.880 °C, which indicated a room temperature reduction of 6.721% with the implementation of VGS.

We have also picked out days on weekends and winter and summer breaks that have the same atmospheric environment with the selected weekdays (similar temperature, humidity, solar radiation amount, *etc.*). The west side had an average temperature of 20.677 °C and the east was 22.453 °C. The temperature reduction was 7.910% without anthropogenic interference.

The results for with/without anthropogenic interference were compared. The average room temperature of a classroom on the west side, without anthropogenic interference was 1.598 °C lower than with anthropogenic interference.

### 3.2. IPMVP

This study used a modified version of option C in the IPMVP to calculate the energy saving for air conditioning in the classroom.

The VGS lowers the temperature by 6.721%. The real room temperature with no VGS is increased by 6.721% of the amount of energy consumed by the air conditioner. The drop in temperature is considered in calculating the amount of energy saved by the VGS.

The steps for calculation of energy saving are:

Step 1: Determine the temperature T of classroom during class time without AC while with VGS:
  (3) $${\text{T}}_{\text{with VGS}}=\text{T of empty classroom}+\text{Anthropogenic interference}\text{1.598}{}^{\circ}\text{C}$$
Step 2: Obtain the T of classroom during class time without AC and without VGS:
  (4) TwithoutVGS=TwithVGS+T reduction by VGS
Step 3: (5) $$\text{Air conditioning load}={\text{T}}_{\text{withoutVGS}}{-\text{T}}_{\text{classroom with AC}}$$
Step 4: Calculate the air conditioning load reduced by VGS:
  (6) $${\text{T}}_{\text{AC load reducing}}={\text{T}}_{\text{withoutVGS}}-{\text{T}}_{\text{withVGS}}$$
Step 5: (7) $$\text{Energy saving}={\text{T}}_{\text{AC load reducing}}*\text{Air conditioner energy saving oefficient}\left(\text{In this study},\text{the energy saving coefficient of the air conditioner is}4.6\%\text{per}{}^{\circ}\text{C}\right)*\text{T}=\text{temperature}$$

Our calculation shows a reduction of approximately 8.308% in the energy consumed by the air conditioner in a day and a maximum of reduction in air conditioner energy consumption of 8.844%. The average reduction is 8.483%, as shown in Table 4.

### 3.3. Verification of Actual Measurements

**Figure 10.**
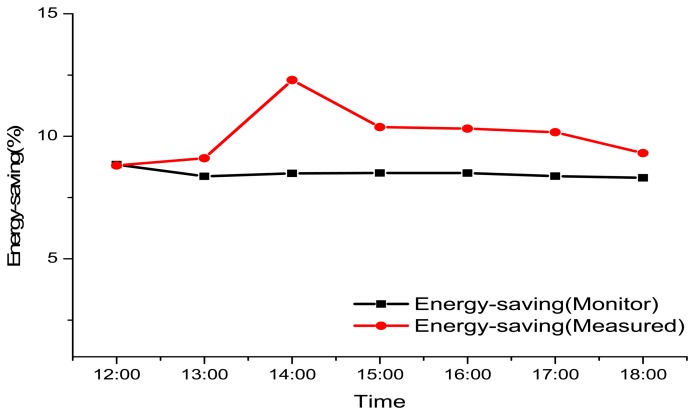
Comparison with real measured results.

Figure 10 shows the days chosen for the model simulation are the same days used in Section 3.2. The measurements and simulation were done by the Department of Energy and Refrigerating Air-Conditioning Engineering of the National Taipei University of Technology. The simulation software used was “Ansys 13 Fluent,” and the results show that the temperature reduction using VGS was 7.046%. The energy saving calculation is seen in Table 5.

### 3.4. Calculation of Electricity Consumption

**Figure 11.**
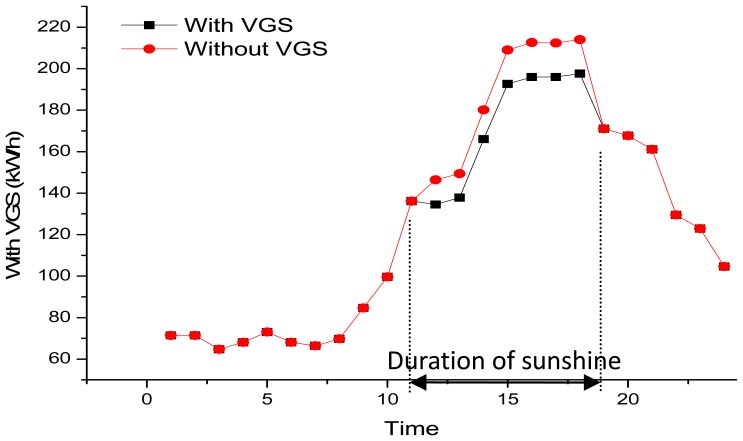
Daily savings in electricity consumption.

In order to verify the IPMVP theory, the actual saving in daily power consumption is recorded. Readings from the west side of the building (with VGS) show that the Sun has a direct impact from noon and until 6:47 pm; during this time span, the energy saved by the VGS is 103.391 kwh/6 h. When the Sun sets, at 6:47 pm, the energy saved by the VGS is zero. The effects are shown in Figure 11.

**Figure 12.**
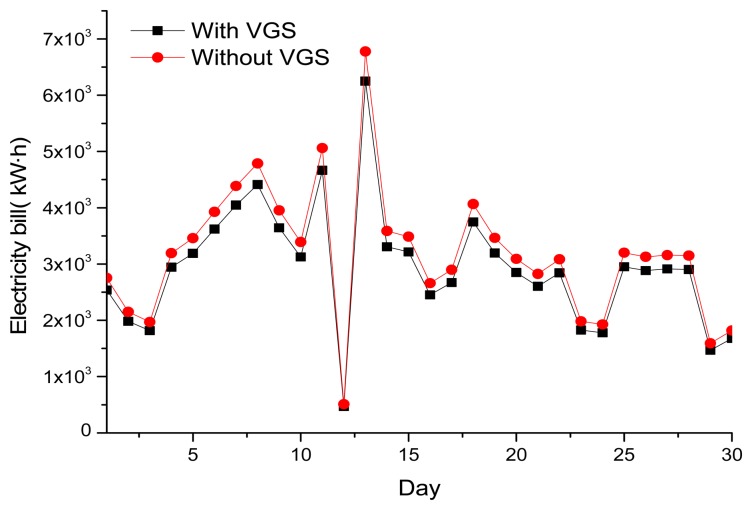
Savings in electricity consumption in one month. (monitoring Date: June 2012).

**Figure 13.**
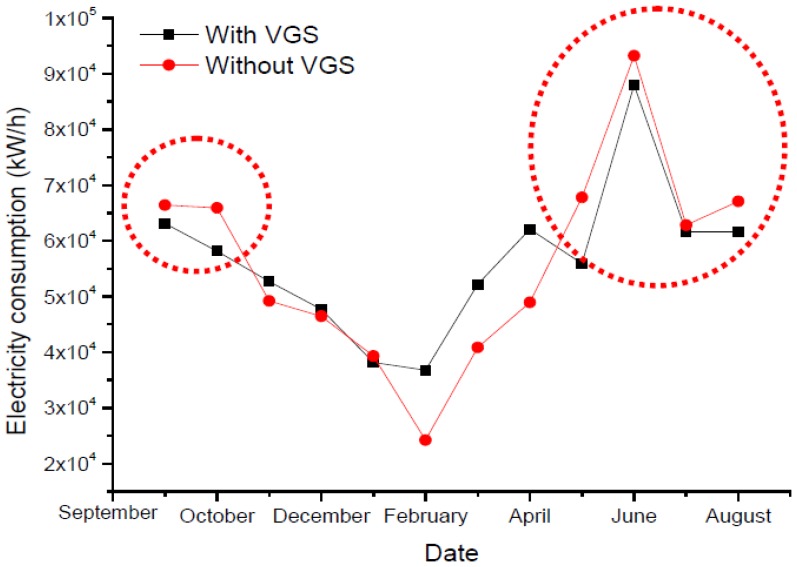
Annual electricity consumption.

The results also show that monthly electricity savings can reach 7,463 kwh/month as illustrated in Figure 12. Figure 13 shows the energy savings for one year (August 2011 ∼ August 2012). VGS has been proven to be effective in energy savings during summer, while in the winter days, the difference is minimal.

### 3.5. Propagation of Uncertainty (Benchmark and Comparison Testing)

**Figure 14.**
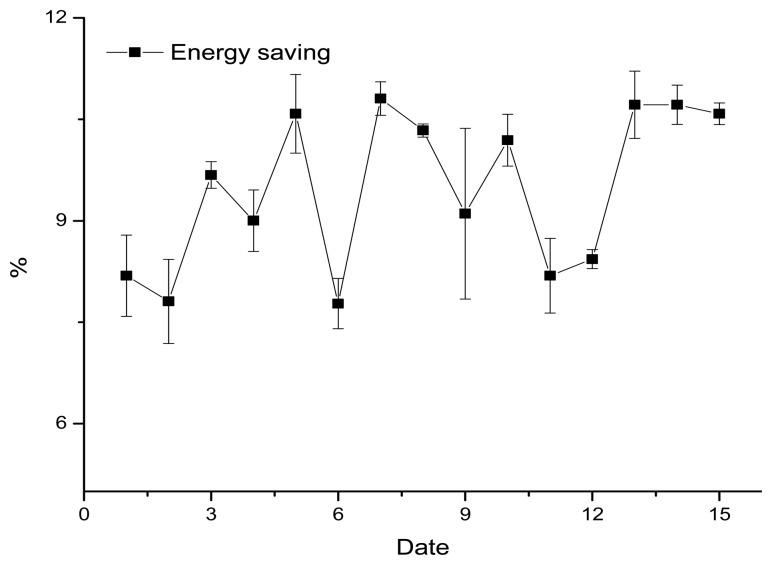
Deviations from the benchmark.

Fifteen days with similar criteria are selected as a benchmark, for comparison purposes. As shown in Figure 14, the data for the days selected differ from the benchmark by −0.924 ∼ +1.141% which is a permissible error. This shows that the method used to calculate energy savings is stable and could be a reference for future studies.

## 4. Conclusions

In conclusion, options A and B in the IPMVP can only measure the amount of energy saved on equipment. Options C and D provide measurement of the overall energy saving, but option C is designed for the analysis of electricity data and is only of use where there are energy savings of more than 10%. Option D requires professional aid and is used for theoretical calculations and simulation models. This study constructs a platform that measures the amount of energy saved, using a modified version of option C as the best solution. The difference between the monitored and theoretical results is only 1.565%. In future, wireless sensors could be used to monitor indoor temperature and the amount of energy saved could be calculated easily and cheaply, using IPMVP option C.

### Outlook (Future Implications)

This paper describes a new method for monitoring and measurement of energy savings. It is hoped that this can be used to calculate energy savings in buildings, and that it will serve as a reference for future studies.

## Figures and Tables

**Table 1.** Summary of options A, B, C and D of the IPMVP and their limitations in implementation for reporting energy saving amounts [15].

**Option**	**Means of Application of the Methodology**	**Implementation Limits**
A	Engineering calculations using spot or short-term measurements, computer simulations, and/or historical data.	The energy saving certifications are aligned on same device, with high cost.
B	Engineering calculations using metered data.	
C	Analysis of utility meter (or sub-meter) data using techniques from simple comparison to multivariate (hourly or monthly) regression analysis.	Precision is not sufficient, for option C, For effective evaluation, the efficiency must be greater than 10%.
D	Calibrated energy simulation/modeling; calibrated with hourly or monthly utility billing data and/or end-use metering.	It must be evaluated by professionals and this method usually requires great skill in the calibration simulation.

**Table 2.** Wireless sensor node model and operating range.

**Item**	**Type**	**Measurement Range**	**Power Supply**	

Relative humidity and temperature sensor	SHT10	20% ∼ 80% ± 4.5%	550 μA at 3 V	
		10 °C ∼ 40 °C ± 0.5 °C		

Photo sensor with signal processing circuits		Accuracy level was ±2% of the reading range, which yielded 1.5 ∼ 4.5 °C uncertainty.	1 mA at 2.5 V	

MEMS flow sensor developed by the lab		0.1 m/s ∼ 0.45 m/s	5 mA at 2.5 V	

Signal processor and RF transmission module	PIC16F526		0.5 ∼ 10 mA at 3.5 V	Nominal power: 20 mW
				Standby power: 1.75 mW
				Transient power max: 35 mW
	
Photovoltaic cell	SC 7035			

**Table 3.** Comparison of IPMVP options and improved version of option C.

***M*&*V Option***	**How Savings Are Calculated?**	***Typical Applications***	***Limits of Applications***
***A. Partially Measured Retrofit Isolation***. Savings are determined by partial field measurement of the energy use of the system(s) to which an ECM was applied, separate from the energy use of the rest of the facility. Measurements may be either short-term or continuous. Partial measurement means that some but not all parameter(s) may be stipulated, if the total impact of possible stipulation error(s) is not significant to the resultant savings.	Engineering calculations using short term or continuous post-retrofit measurements and stipulations.	Lighting retrofit where power draw is measured periodically. Operating hours of the lights are assumed to be one half hour per day longer than store open hours.	Option A can only measure the amount of energy saved on equipment.
***B. Retrofit Isolation***. Savings are determined by field measurement of the energy use of the systems to which the ECM was applied, separate from the energy use of the rest of the facility. Short-term or continuous measurements are taken throughout the post-retrofit period.	Engineering calculations using short term or continuous measurements.	Application of controls to vary the load on a constant speed pump using a variable speed drive. Electricity use is measured by a kWh meter installed on the electrical supply to the pump motor. In the base-year this meter is in place for a week to verify constant loading. The meter is in place throughout the post-retrofit period to track variations in energy use.	Option A can only measure the amount of energy saved on equipment.
***C. Whole Facility*** Savings are determined by measuring energy use at the whole facility level. Short-term or continuous measurements are taken throughout the post-retrofit period.	Analysis of whole facility utility meter or sub-meter data using techniques from simple comparison to regression analysis.	Multifaceted energy management program affecting many systems in a building. Energy use is measured by the gas and electric utility meters for a twelve month base-year period and throughout the post-retrofit period.	Option C is designed for the analysis of electricity data and is only of use where there are energy savings of more than 10%. Also an observation and data collection of over a year is required.
***D. Calibrated Simulation*** Savings are determined through simulation of the energy use of components or the whole facility. Simulation routines must be demonstrated to adequately model actual energy performance measured in the facility. This option usually requires considerable skill in calibrated simulation.	Energy use simulation, calibrated with hourly or monthly utility billing data and/or end use metering.	Multifaceted energy management program affecting many systems in a building but where no base-year data are available. Post-retrofit period energy use is measured by the gas and electric utility meters. Base-year energy use is determined by simulation using a model calibrated by the post-retrofit period utility data.	Option D requires professional aid and is used for theoretical calculations and simulation models. Option D requires a year of historical data for comparison.
***Improved version of option C:*** Savings are determined by measuring energy use at the whole facility level. Short-term or continuous measurements are taken throughout the post-retrofit period, while the environment variables are also taken into consideration.	Electricity consumption of the building is recorded, while the change in room temperature and air-conditioning used is monitored by a sensor platform. The energy saved and energy efficiency is then calculated.	Monitoring energy usage and actual energy-saving effect for a whole building.	Improved version of option C provides measurement of the overall energy saving, with much less limitations compared to options C and D. It is also easier to calculate.

**Table 4.** Energy Saving calculated by IPMVP Option C (improve) in a day (25 June 2012).

**Time**		12:09	13:09	14:09	15:09	16:09	17:09	18:09
**Room temperature (w/air-condition)**		27.00	26.30	25.52	25.33	25.00	24.82	24.60
**Normal calculation**	Room temperature w/o air-conditioning (°C)	28.61	27.06	27.46	27.51	27.50	27.09	26.88
	Air condition dosage (°C)	1.61	0.76	1.94	2.18	2.50	2.27	2.28
**Improve option C**	Room temperature w/o air-conditioning (Plus anthropogenic factor) (°C)	30.21	28.66	29.06	29.11	29.10	28.69	28.47
	Air condition dosage (°C)	3.21	2.36	3.54	3.78	4.10	3.87	3.87
	Fixed room temperature w/o air conditioning (Plus reduced degree by VGS) (°C)	32.13	30.47	30.90	30.96	30.95	30.51	30.28
	Air condition dosage (°C)	5.13	4.17	5.38	5.63	5.95	5.69	5.68
Savings in air conditioning dosage by VGS (°C)		1.92	1.82	1.85	1.85	1.85	1.82	1.81
Saved air conditioning (%)		8.844	8.364	8.489	8.504	8.501	8.375	8.308

**Table 5.** Value of the temperature tested (measurement date: 25 June 2012).

**Time**		**PM12**	**PM13**	**PM14**	**PM15**	**PM16**	**PM17**	**PM18**	**Average**
External wall temperature	Before	30.92	31.16	32.50	31.17	30.66	30.56	30.09	31.00
	Be hide	29.00	29.19	29.82	28.92	28.42	28.35	28.07	28.82
Cooling effectiveness (°C)		1.92	1.97	2.68	2.25	2.24	2.21	2.02	2.18
Energy efficiency (%)		8.811	9.105	12.294	10.374	10.311	10.166	9.292	10.048
